# Design of an Inkjet-Printed Rotary Bellows Actuator and Simulation of its Time-Dependent Deformation Behavior

**DOI:** 10.3389/frobt.2021.663158

**Published:** 2021-06-10

**Authors:** Gabriel Dämmer, Michael Lackner, Sonja Laicher, Rüdiger Neumann, Zoltán Major

**Affiliations:** ^1^ Institute of Polymer Product Engineering, Johannes Kepler University Linz, Linz, Austria; ^2^ Advanced Development Control and Robotics, Festo SE and Co. KG, Esslingen, Germany

**Keywords:** bellow actuator, printed robotics, design for additive manufacture, multi-material 3D printing, soft pneumatic actuator, time-dependent materials, printed elastomer, polyjet elastomers

## Abstract

State-of-the-art Additive Manufacturing processes such as three-dimensional (3D) inkjet printing are capable of producing geometrically complex multi-material components with integrated elastomeric features. Researchers and engineers seeking to exploit these capabilities must handle the complex mechanical behavior of inkjet-printed elastomers and expect a lack of suitable design examples. We address these obstacles using a pneumatic actuator as an application case. First, an inkjet-printable actuator design with elastomeric bellows structures is presented. While soft robotics research has brought forward several examples of inkjet-printed linear and bending bellows actuators, the rotary actuator described here advances into the still unexplored field of additively manufactured pneumatic lightweight robots with articulated joints. Second, we demonstrate that the complex structural behavior of the actuator’s elastomeric bellows structure can be predicted by Finite Element (FE) simulation. To this end, a suitable hyperviscoelastic material model was calibrated and compared to recently published models in a multiaxial-state-of-stress relaxation experiment. To verify the material model, Finite Element simulations of the actuator’s deformation behavior were conducted, and the results compared to those of corresponding experiments. The simulations presented here advance the materials science of inkjet-printed elastomers by demonstrating use of a hyperviscoelastic material model for estimating the deformation behavior of a prototypic robotic component. The results obtained contribute to the long-term goal of additively manufactured and pneumatically actuated lightweight robots.

## Introduction

**FIGURE 1:**
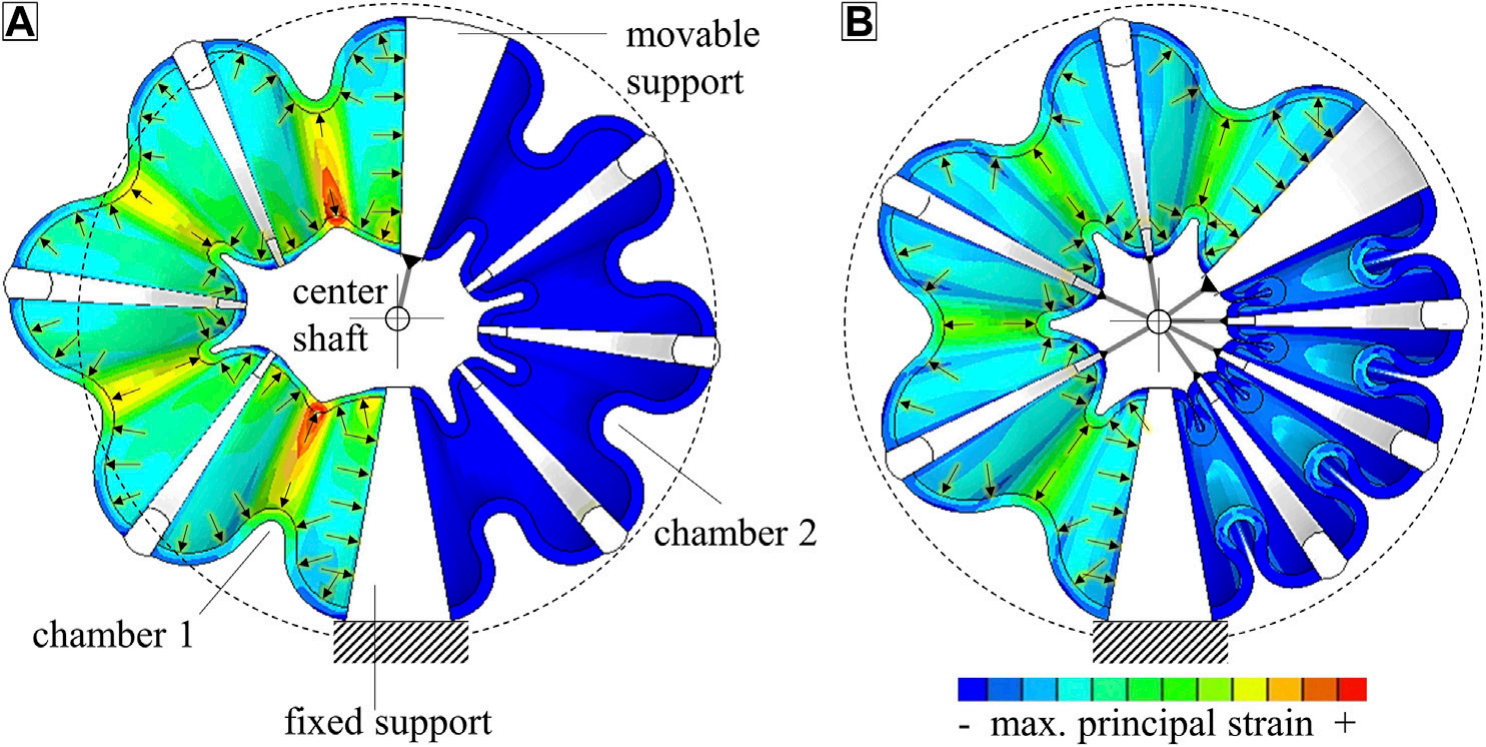
Schematics and typical strain distributions in rotary bellows actuators without **(A)** and with **(B)** constrained motion of the elastomeric bellows chambers. Applying a pressure difference between chambers 1 and 2 causes the bellows actuator to rotate. Constraining the radial displacement of the bellows structure **(B)** improves performance, inhibits excessive deformation and results in more homogeneous distribution of strain than in an unconstrained design **(A)**.

Additive Manufacturing (AM) facilitates quick and cost-efficient iterations of design, manufacturing and testing, and its application has become common practice for simple prototyping purposes. State-of-the-art AM technologies can already process multiple polymeric materials into functionally integrated, and thus potentially compact, lightweight components that require little assembly. Against this background, it seems worthwhile to consider polymer-based AM processes for manufacturing of lightweight robots in the long-term. Lightweight robotics is a growing subfield of robotics which addresses novel manipulator systems that operate in flexible working environments. The resulting requirements of compactness, high power-to-weight ratio and cost efficiency create conflicting goals regarding the mechanical design of future lightweight robots (Gealy et al., 2019). However, AM has not yet caught up with conventional technologies in the manufacturing of safety-relevant or functionally critical components. What seems to hinder use of AM is that the complex mechanical behavior of AM polymers is insufficiently known and seems difficult to predict due to a lack of advanced structural simulations and prototyping use cases. The AM of suitable actuators for lightweight robots seems to be particularly challenging but the use of printed pneumatic bellows actuators presents a promising approach and could become a decisive factor for the success of pneumatic lightweight robots in the long-term. Bellows actuators overcome some of the difficulties in printing conventional pneumatic actuation systems (MacCurdy et al., 2016) which require tight tolerances and smooth surfaces and the structural behavior of bellows actuators can easily be modified by varying shape and material (Dämmer et al., 2019a). In bellows actuators, actuation results from expansion of a deformable chamber due to pressurization. Structural forces are guided along curved load paths—the typical bellows shape—which, in combination with use of compliant materials such as elastomers, minimizes structural stiffness in the direction of actuation. Nowadays, commercial lightweight robots are articulated and thus require actuators that create controllable torque and motion about a defined axis of articulation. This can be achieved by using the bellows principle, as shown in two example schematic designs for rotary bellows actuators in Figure 1. Two curved deformable bellows chambers are arranged about a central axis and are connected to one fixed and one movable support. Applying pressure to one of the bellows chambers causes its expansion and thus rotation of the movable support, which is typically connected to a drive shaft. The fixed support can be connected to a housing or surrounding structure. However, without further constraining the deformation of the bellows structures, pressurization can lead to excessive deformation and strains, as shown schematically in Figure 1A. To address this problem, various mechanisms have been invented that force the bellows structures motion to follow a circular path. As in the example given in Figure 1B, additional tension elements, such as plates (Stoll and Schlötzer, 2008) or strips of fabric (Steffen, 2006) can be used to attach the bellows chambers to a center shaft. The deformable bellows chambers can also be clamped directly to the center of rotation (Bousso, 1970). Kaminski and Knubben (Kaminski and Knubben, 2012) used a bellows design that impedes excessive radial displacement and achieved rotary motion by means of a kinematic mechanism. Other approaches (Kargov et al., 2008; Gaiser et al., 2010) use hinges to transform the expansion of linear bellows actuators into rotation. In some mechanisms, excessive radial displacement and buckling of the curved bellows segments are prevented by specific design and by material selection (Sigmon and Charlotte, 1976). In other mechanisms, the bellows chambers are encapsulated within a cylindrical housing, as demonstrated in a hydrodynamic shock-absorbing device (Ficht and Vilsmeier, 1987).

**FIGURE 2:**
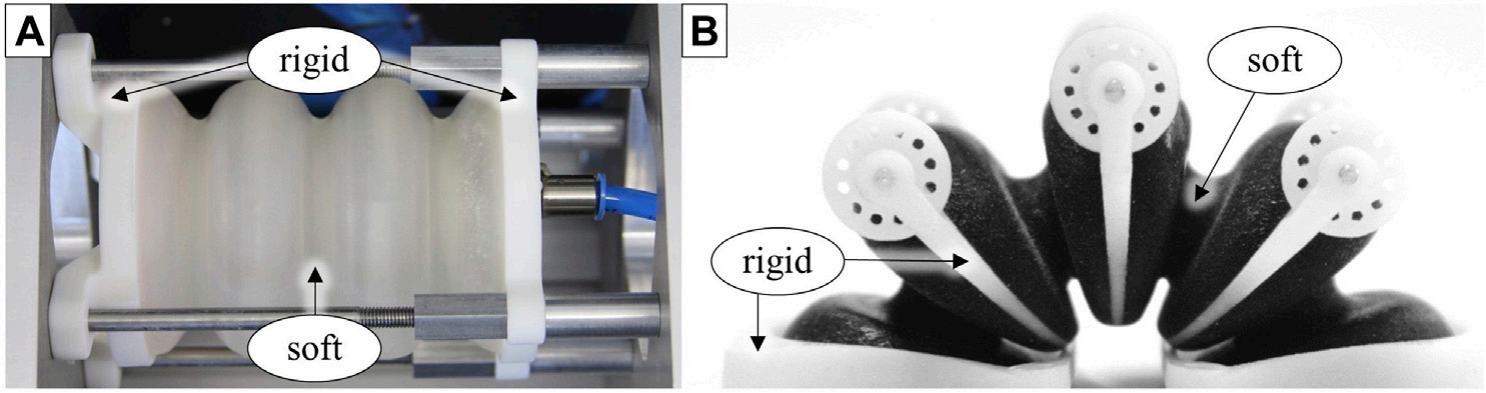
Bellows actuators manufactured by 3D inkjet printing of thermosetting soft and rigid materials. **(A)**: Linear actuator for investigating the effects of shape and material variations on structural behavior and fatigue (Dämmer et al., 2019a). **(B)**: Rotary bellows chamber for actuation of a lightweight gripper (Dämmer et al., 2019b).

Researchers have already demonstrated that rotary bellows actuators similar to those shown in Figure 1 can be used to build functional articulated robots (Ivlev, 2009; Taghia et al., 2012). However, research published in this context, of which a detailed overview exists (Gaiser et al., 2012), has not focused on AM. Although AM of elastomers in general (Herzberger et al., 2019) and of soft actuators in particular (Zolfagharian et al., 2016) has been reviewed, to the best of our knowledge, multi-material AM technologies have not yet been used to produce rotary bellows actuators. In 3D inkjet printing, which is widely used in soft robotics, droplets of acrylic photocurable ink are deposited *via* piezo nozzles, cured by infrared light and accumulated to objects of desired shapes (Schmitt et al., 2018; Herzberger et al., 2019). Programming parallel deposition of droplets of different materials allows 3D multi-material objects to be produced (Schmitt et al., 2018). These may consist of materials with different physical properties, such as color (Scharff et al., 2018), hardness (Stratasys, Ltd., 2017; Dämmer et al., 2019a) and electrical conductivity (Saleh et al., 2017); even ceramic-filled inks for abrasion-resistant components are being developed (Graf et al., 2020). However, 3D inkjet printing is currently used predominantly to produce monolithic multi-material parts with soft and rigid features. PolyJet, the most common 3D inkjet technology, processes thermosetting inks that cure either to elastomeric solids, such as TangoPlus (T+) and Agilus30 (A30) (Stratasys, Ltd. 2017), or to rigid solids, such as the materials of the Vero family (Stratasys, Ltd. 2017). Although multi-material AM of rotary bellows actuators has not been studied, PolyJet technology has been used to produce mono- and multi-material linear and bending bellows actuators. Scharff et al. (Scharff et al., 2018) used color sensors to measure the deformation of specially designed multi-color bending bellows actuators printed from A30 and Vero materials of different colors. Zhang et al. (Zhang et al., 2019) integrated a shape-memory polymer layer into A30 bending actuators to achieve adjustable structural stiffness. Drotman et al. (Drotman et al., 2017; Drotman et al., 2018) combined linear actuators to bending-actuator modules that were used for a soft manipulator and printed from a material with a Shore A hardness of 70. Blumenschein and Mcngüç (Blumenschein and Mcngüç, 2019) combined A30 linear actuators to parallel delta mechanisms in order to achieve higher-degree-of-freedom motions. Du Pasquier et al. (Du Pasquier et al., 2019) presented A30 linear and bending bellows actuators that could be used as building blocks for soft robots. Zhang et al. (Zhang et al., 2020) integrated bellows actuators manufactured by printing A30 and Vero materials to actuate the fingers of an anthropomorphic hand. These materials were also used by Sheng et al. (Sheng, et al., 2020) to realize a crawling robot with deformable bellows structure. Dämmer et al. (Dämmer et al., 2019a) used A30 and T+ in combination with Vero materials to produce the linear bellows actuators shown in Figure 2A and investigated the effects of material and shape variations on their fatigue and deformation behavior. In a subsequent publication, Dämmer et al. (Dämmer et al., 2019b) transferred their findings to rotary bellows chambers of similar materials and demonstrated their integration into a robotic lightweight gripper; Figure 2B shows the chamber design, which comprises four elastomeric bellows segments connected by three rigid frames and a roller guide system that prevents excessive deformation. The specific shape of the bellows segments is based on conceptual Finite Element (FE) simulations (Dämmer et al., 2019b). With the objective of strain homogenization, local wall thicknesses were tuned within multiple iterations of design and analysis. The work shown in this paper uses a similar chamber shape and guide system and presents a universal actuator design with a wide range of applications.

Generally, the complex mechanical behavior of PolyJet-printed elastomers in advanced prototypes is challenging: Numerous publications have described non-linear stress-strain relations (Liljenhjerte et al., 2016; Slesarenko and Rudykh, 2018; Dämmer et al., 2019a; Du Pasquier et al., 2019; Morris et al., 2019) and time-dependent mechanical behavior (Blanco et al., 2014; Kundera and Bochnia, 2014; Adamczak and Bochnia, 2016; Reichl and Inman, 2018). In order to model the constitutive behavior of these materials, researchers have recently calibrated hyperviscoelastic material models by means of uniaxial calibration experiments. These models assume both isotropy and incompressibility. Slesarenko and Rudykh (Slesarenko and Rudykh, 2018) described a pronounced rate-dependent behavior of T+ and combinations of T+ and Vero material, and calibrated a hyperviscoelastic model using uniaxial tensile experiments at various strain rates. The data provided in that paper (Slesarenko and Rudykh, 2018) can be used to define a Yeoh strain energy potential with a three-term Prony series in common Finite Element Analysis (FEA) software. However, modeling of A30 is of particular interest, since it has shown superior fatigue life compared to T+ (Dämmer et al., 2019a) and is frequently used in soft robotics (e.g., Scharff et al., 2018; Blumenschein and Mcngüç, 2019; Zhang et al., 2019; Zhang et al., 2020). Very recently, researchers have investigated the viscoelastic material properties of A30 and calibrated material models accordingly. Dykstra et al. (Dykstra et al., 2019) studied the effect of viscoelasticity on the behavior of elastomeric metamaterials printed from A30. Based on uniaxial stress relaxation experiments, a hyperviscoelastic material model was calibrated. The data provided (Dykstra et al., 2019) can be used to define a Neo-Hookean strain energy potential and a three-term Prony series. To the best of our knowledge, the most comprehensive series of mechanical experiments and analyses of A30 and T+ to date were provided by Abayazid and Ghajari (Abayazid and Ghajari, 2020). Uniaxial large-strain tension and compression experiments were performed to investigate the time-independent material behavior. Furthermore, time dependency was studied and modeled by means of uniaxial high-frequency and relaxation loadings. The material model parameters provided (Abayazid and Ghajari, 2020) can be used directly to define a three-term Ogden strain energy potential in combination with a five-term Prony series.

Two main problems emerge when summarizing the state of research related to multi-material 3D inkjet printing of rotary bellows actuators. First, although some examples of conventionally manufactured rotary bellows mechanisms are available, multi-material AM technologies, such as 3D inkjet printing, have been applied in a single study only (Dämmer et al., 2019b). Hence, there was no broad base on which to build our research into multi-material AM of rotary bellows actuators or to assess their relevance to prototyping and production of future robotic systems. Second, recent research has addressed the constitutive modeling of inkjet-printed elastomers, including non-linear stress-strain relations and time dependency. In this context, researchers have calibrated hyperviscoelastic material models based on laboratory specimens and provided model parameters for direct input into commercial FEA software. However, the suitability of these models for accurate simulation of the time-dependent behavior of geometrically complex bellows structures has yet to be demonstrated. The research presented in the following addresses both problems. We present a novel, functionally integrated rotary bellows actuator that is suitablefor 3D inkjet printing to a make an early foray into this unexplored field of actuators. In preparation for the simulation of its time-dependent structural behavior, we calibrated a suitable hyperviscoelastic material model, which we validated and compared to material models from recent publications (Slesarenko and Rudykh, 2018; Dykstra et al., 2019; Abayazid and Ghajari, 2020) using a bellows stress relaxation experiment as published by Dämmer et al. (Dämmer et al., 2019a). The time-dependent structural behavior of our rotary actuator was then investigated in experiments and corresponding FE simulations. Thus, the practical relevance of time dependency and applicability of the proposed material models were assessed.

## Actuator Design

In this section, we present a novel elastomeric rotary bellows actuator that is suitable for 3D inkjet printing technology. Following a description of the actuator’s components and principle of operation, aspects of functional integration are discussed.

### Operating Principle and Design Overview

**FIGURE 3:**
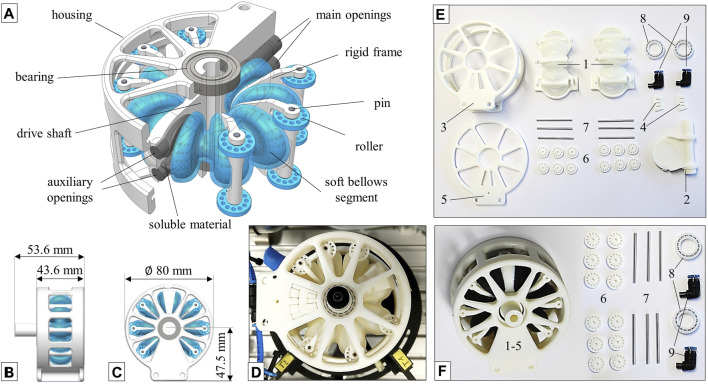
Novel rotary bellows actuator design for multi-material AM with soft bellows segments and rigid features. Components and design features are identified in the sectional CAD view **(A)**, and the actuator’s main dimensions are given in **(B,C)**. Applying a pressure difference between the bellows chambers results in angular motion of the actuator which is guided by a roller system **(D)**. A “partly integrated” variant **(E)** consists of nine different components, each of which can be replaced individually. In a “highly integrated” variant **(F)**, the actuator’s main components (1–5) are integrated into a single multi-material part to minimize assembly.

Figure 3A shows a sectional CAD view of the novel rotary actuator. In accordance with the functional principle described in *Introduction*, two pneumatic bellows chambers are arranged antagonistically around a rotatable drive shaft. Each chamber forms a separate volume and connects the moving drive shaft to a fixed housing. The bellows chambers are built by serial connection of four soft elastomeric bellows segments interleaved with three rigid frames. Each rigid frame holds two rollers and a metal pin. The rollers can rotate freely about the pins and rest against the housing. Inside the housing, annular grooves prevent undesired shifting of the rollers. A set of bearings guides the relative motion between drive shaft and housing. Each chamber can be pressurized *via* an internal air duct. The housing has a diameter of 80 mm and a height of 43.6 mm and the actuator’s main dimensions are given in Figures 3B,C. Torque is generated by applying a pressure difference between the two chambers. As the expansion is directed by the roller guide system and bearings, the shaft rotates about the main axis, as illustrated in Figure 3D and in accordance with Figure 1B. At a pressure of 9.0 kPa, the actuator produces torques of up to 20 Nmm at 0° and can reach angles close to 24°. Larger pressure can be applied to increase torque and angular displacement but will reduce the lifespan of the bellows structure. The assembled actuator weighs 105 g.

### Case Study: Functional Integration

Two variants of the novel actuator were developed that provide the same general functionality but differ in the degree of functional integration. Both variants are shown in Figure 3 and are hereafter referred to as “partly integrated” (E) and “highly integrated” (F), respectively. The components required to assemble the partly integrated version are presented in Figure 3E. The bellows chambers 1) are fully integrated multi-material components, each of which consists of four elastomeric bellows segments, three frames and flanges for attachment to the drive shaft 2) and bottom housing 3). Attachment clips 4) are used in combination with the housing cover 5) to hold the bellows chambers in place. The assembly is completed with rollers 6), pins 7), bearings 8) and pneumatic connectors 9). This (partly integrated) version allows replacement of individual bellows chambers in a series of fatigue experiments, which will be presented in a future publication. Figure 3F shows the components required to assemble the highly integrated actuator. In this variant, the bellows chambers, the mounting clips, the housing and the drive shaft are combined to a single part, as shown in Figure 3F. Only mounting of the rollers 6), pins 7), bearings 8) and pneumatic connectors 9) are required to complete the actuator’s assembly. By modifying the partly integrated version, we were thus able to combine seven parts into one highly integrated component (Figure 3F, left) without sacrificing any function or performance. This is made possible by effective exploitation of the advantages specific to 3D inkjet technology, such as combined printing of soft and hard materials and printing of complex overhangs. However, in 3D inkjet printing, overhangs are realized using support material and measures had to be taken to allow for removal of support material inside the pressure chambers after printing. We used soluble support material, increased the diameter of the main openings to 8 mm and added auxiliary openings of 6 mm in diameter to the movable flange as shown in Figure 3A. These openings were used to flush the bellows chambers and carefully remove the support material which turned out to be a feasible but laborious process.

Both actuators were manufactured using PolyJet technology and Vero materials for the structural parts. For the highly integrated variant, A30 was chosen as a soft material. For the partly integrated variant, a combination of A30 and Vero with a Shore A hardness of 70 was used. All actuators were printed with the main axis perpendicular to the printing plane. While earlier designs of rotary bellows mechanisms were based on multiple components (Bousso, 1970; Ficht and Vilsmeier, 1987; Stoll and Schlötzer, 2008) or consisted largely of a single material (Kaminski and Knubben, 2012), the main component of the highly integrated actuator is manufactured as one monolithic multi-material structure.

## Modeling the Mechanical Behavior of Inkjet-Printed Elastomers

This section presents the development of a structural material model for inkjet-printed elastomers suitable for implementation in commercial FEA software, such as Abaqus (Dassault Systèmes SE) and other nonlinear solvers. We describe the initial tensile experiments, selection and theoretical summary of a suitable constitutive model, and the calibration procedure for obtaining the model parameter values. Finally, the accuracy of the material model was assessed by comparison of a bellows relaxation experiment with corresponding FE simulations.

### Mechanical Behavior of Inkjet-Printed Elastomers

**FIGURE 4:**
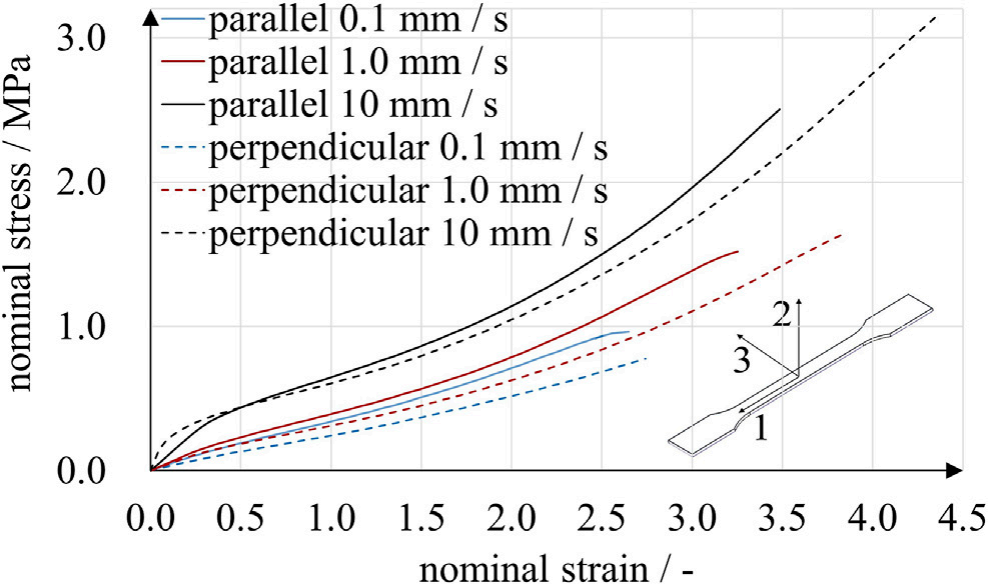
Uniaxial tensile experiments with A30 specimens. Two print directions and three loading rates were compared. Specimens printed parallel to the 1-3-plane showed stiffer behavior than those printed perpendicular to the 1-3-plane. Under the given experimental conditions, the stresses observed for a particular strain were affected more by loading rate than by print direction.

**FIGURE 5:**
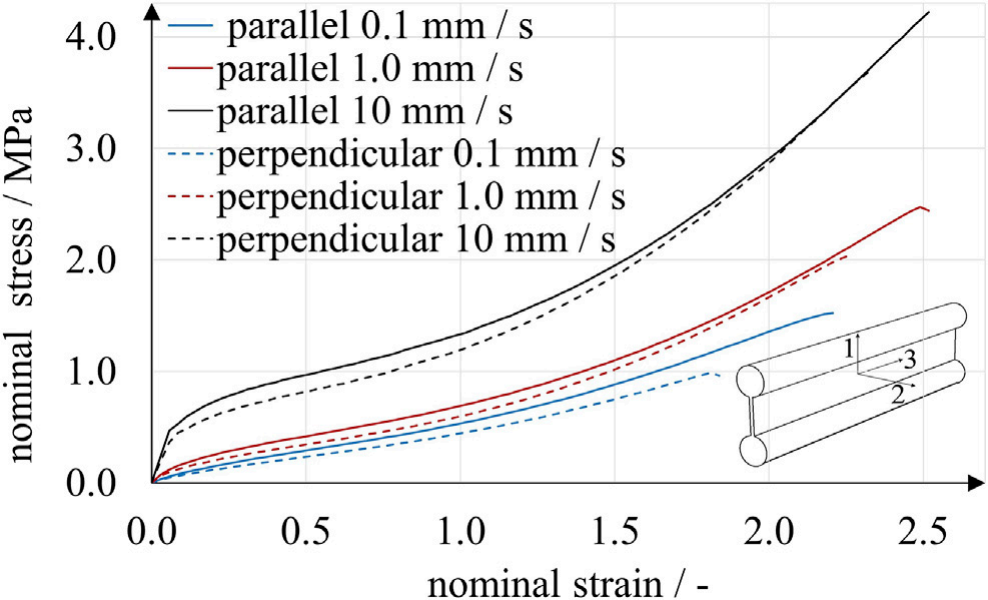
Planar tensile experiments with A30 specimens. Two print directions and three loading rates were compared. While the effect of loading rate on stress was considerable, print direction had a distinct but smaller effect.

In order to examine the basic material behavior of the inkjet-printable elastomers A30 and T+ (Stratasys, Ltd., 2017), uniaxial and planar tensile experiments were performed. For the uniaxial tensile experiments, a specimen shape according to DIN EN ISO 527–2, type 5 A (DIN EN ISO 527-2:2012, 2012) with a nominal thickness of 2 mm was chosen. In the uniaxial tensile experiment, the deformation of the center region of the specimen is described by the principal stretches $\lambda_{j}$ (*j* = {1, 2, 3}) as *λ*_1_ = *l*_1_/*l*_1,0_ and *λ*_2_ = *λ*_3_ = $\lambda_{1}^{-1/2}$, where *l*_1_ and *l*_1,0_ are the current and the initial lengths of the relevant specimen sector in the 1-direction. For the planar tensile experiments, we chose rectangular plate specimens with a thickness of 1 mm and a large aspect ratio of 10 mm/100 mm to achieve the desired state of deformation described by *λ*_1_ = *l*_1_/*l*_1,0_, *λ*_2_ = 1/*λ*_1_ and *λ*_3_ = 1. A detailed description of specimen geometry and background information on planar tensile experiments were given by Çakmak et al. (Çakmak et al., 2014). Our experimental setup comprised a linear actuator for monotonic experiments (Bose Corp., ElectroForce Systems Group, MN, United States), a WMC-25 load cell (Interface Inc. AZ, United States) and a controller unit and workstation with WinTest® DMA software (Bose Corp.) All specimens were printed at cirp GmbH (Römerstraße 8, 71,296 Heimsheim, Germany). In the experiments, specimens were elongated at loading rates of 0.1 mm/s, 1.0 mm/s and 10.0 mm/s until rupture, and the reaction forces were measured. Nominal strain rates were calculated to be 0.005 1/s, 0.05 1/s and 0.5 1/s for the uniaxial experiments and 0.01 1/s, 0.1 1/s and 1.0 1/s or the planar experiments. All experiments were performed at an ambient temperature of 21°C. In Figures 4, 5, the results of monotonic uniaxial and planar tensile experiments are plotted, respectively. Sketches of the specimen geometries are shown together with body-fixed coordinate systems that indicate the principal directions. Specimens printed in the 2-direction are hereafter referred to as “parallel-printed”, as layers and printing plane are in parallel orientation. Specimens printed in the 1-direction are referred to as “perpendicular-printed”, following the same logic. Figures 4, 5 plot nominal stress over nominal strain, relative to the 1-axis, for various loading rates and the two print directions. Each curve is an average of one to three experiments, and each experiment was performed using a pristine specimen. The end of each curve indicates occcurrence of the first rupture.

The results obtained from uniaxial and planar tensile experiments show a typical mechanical elastomer behavior with significantly non-linear stress-strain relation. Up to a deflection point at a nominal strain of approximately 0.5, the curves are degressive, followed by a region of positive curvature until rupture. For all loading rates, the rupture strains were above 2.5 and 1.5 for the uniaxial and planar experiments, respectively. In accordance with Abayazid and Ghajari (Abayazid and Ghajari, 2020), the imposed strains correlated with higher stresses for specimens printed parallel to the printing plane. This observation is valid for all results except for strains below 0.5 at the highest loading rate under uniaxial stress. Apparently, stress is affected much more by strain rate than by build direction, at least under these specific experimental conditions. For example, in the uniaxial experiments using perpendicular-printed specimens at a nominal strain of 0.5, nominal stresses ranged from 0.13 to 0.44 MPa for loading rates of 0.1 mm/s and 10 mm/s, respectively. The absolute difference of nominal stresses increased with nominal strain. At a nominal strain of 2.0, nominal stresses ranged from 0.53 to 1.06 MPa for loading rates of 0.1 mm/s, 1.0 mm/s and 10 mm/s.

In accordance with recent publications (Slesarenko and Rudykh, 2018; Dykstra et al., 2019; Abayazid and Ghajari, 2020), we modeled the non-linear time-independent mechanical behavior and imposed time dependency in a subsequent step, as described below.

### Constitutive Models for Inkjet-Printed Elastomers

Hyperelasticity describes a fully elastic, non-linear stress-strain relation and is in many cases a good approximation of the large-strain behavior of elastomeric materials (Ogden, 1997; Bergström, 2015). Hyperelastic behavior is typically modeled by a strain energy function *U* that relates the stored strain energy per reference volume to the principal stretches $\lambda_{j}$ (Ogden, 1997; Dassault Systèmes Simulia Corp, 2014a). In this section, the Neo-Hooke, Yeoh and Ogden strain energy potentials are introduced briefly as starting points for model calibration and the structural simulations described in the subsequent sections. Detailed information on these material models and their implementation in FE software can be found in according literature (Ogden, 1997; Dassault Systèmes Simulia Corp, 2014a; Dassault Systèmes Simulia Corp, 2014b; Bergström, 2015). The Neo-Hooke and Yeoh forms include the principal stretches in the form of the first invariant of the left Cauchy-Green deformation tensor $I_{1}$ given by

$$I_{1}=\lambda_{1}^{2}+\lambda_{2}^{2}+\lambda_{3}^{2}.$$ (1)

The incompressible form of the Neo-Hooke strain energy potential is given by

$$U=C_{10}\left(I_{1}-3\right),$$ (2)

where *C*_10_ is a material parameter. A slightly different formulation of the Neo-Hooke strain energy potential was used by Dykstra et al. (Dykstra et al., 2019), who additionally provided the required material parameter. Slesarenko and Rudykh (Slesarenko and Rudykh, 2018) used a two-term Yeoh model that contains two model parameters, *C*_10_ and *C*_20_, and can be expressed as (Dassault Systèmes Simulia Corp, 2014b):

$$U=C_{10}\left(I_{1}-3\right)+C_{20}{\left(I_{1}-3\right)}^{2}.$$ (3)

In the Ogden form, which was also used by Abayazid and Ghajari (Abayazid and Ghajari, 2020), *U* is directly expressed as a function of the stretches $\lambda_{j}$. With the moduli *μ*_*i*_ and the non-dimensional material constants *n* and *α*_*i*_, the Ogden strain energy potential (Dassault Systèmes Simulia Corp, 2014-2) is given by

$$U=\;\sum_{i=1}^{n}\frac{2\mu_{i}}{\alpha_{i}^{2}}\left(\lambda_{1}^{\alpha_{i}}+\lambda_{2}^{\alpha_{i}}+\lambda_{3}^{\alpha_{i}}-3\right).$$ (4)

Derivation of the strain energy potentials with respect to stretches yields the corresponding stresses. For the Ogden model (Eq. 4), the stresses σ_*uniax*_ and σ_*planar*_ for uniaxial and planar tension are given, respectively, by (Dassault Systèmes Simulia Corp, 2014b):

$$\sigma_{\mathrm{uniax}}=\frac{\partial U}{\partial \lambda_{1}}=\sum_{i=1}^{n}\frac{2\mu_{i}}{\alpha_{i}}\left(\lambda_{1}^{\alpha_{i}-1}-\lambda_{1}^{-0.5\alpha_{i}-1}\right);$$ (5)

$$\sigma_{\mathrm{planar}}=\frac{\partial U}{\partial \lambda_{1}}=\overset{n}{\underset{i=1}{\sum }}\frac{2\mu_{i}}{\alpha_{i}}\left(\lambda_{1}^{\alpha_{i}-1}-\lambda_{1}^{-\alpha_{i}-1}\right).$$ (6)

To account for the significant strain-rate dependency observed in the tensile experiments (Figures 4, 5), we used Prony series expansions that describe a time-dependent decay of the material model parameter values $\mu_{i}$ as shown in Eq. 7. Prony series are the mathematical equivalent of generalized Maxwell elements and are typically used to model linear viscoelastic behavior (Ogden, 1997; Dassault Systèmes Simulia Corp, 2014a). Expressing the shear-related time-dependent Ogden coefficients $\mu_{i}^{\text{R}}\left(t\right)$ by means of Prony series yields

$$\mu_{i}^{R}\left(t\right)\;=\mu_{i}[1-\;\overset{m}{\underset{k=1}{\sum }}g_{k}\left(1\;-\;e^{-\frac{t}{\tau_{k}}}\right)],$$ (7)

with the coefficients of the instantaneous time-independent material behavior $\mu_{i}$, the Prony parameters $g_{k}$ and relaxation times $\tau_{k}$. Eq. 7 has the limiting values $\mu_{i}^{R}\left(0\right)$ = $\mu_{i}$ and $\mu_{i}^{R}\left(\infty \right)=$ $\mu_{i}\;[1-\;\overset{m}{\underset{k=1}{\sum }}g_{k}],$ and thus the initial behavior is fully described by the initial moduli *µ*_*i*_, while long-term behavior is captured by the sum of Prony parameters. Combining **Eqs. 7, 8** yields the time-dependent Ogden form of the strain energy potential *U*(*t*) for incompressible isotropic materials as implemented in Abaqus/CAE (Dassault Systèmes Simulia Corp, 2014a; Dassault Systèmes Simulia Corp, 2014b). By performing similar operations, time dependency can be introduced into the other models described above (Eqs. 2, 3) and as demonstrated in recent publications (Slesarenko and Rudykh, 2018; Dykstra et al., 2019; Abayazid and Ghajari, 2020).

### Material Model Calibration

**FIGURE 6:**
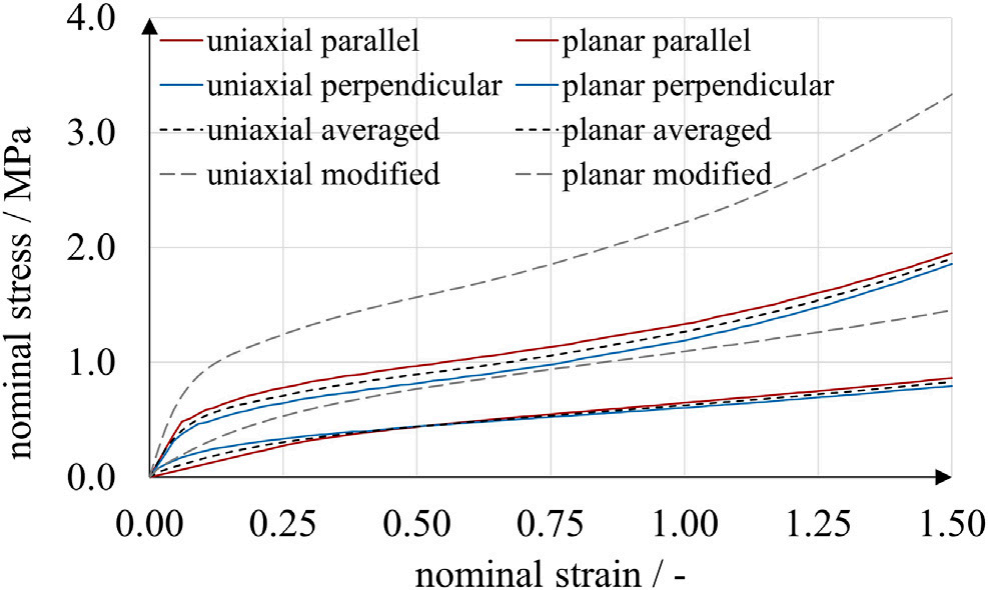
Results of uniaxial and planar tensile experiments used for the calibration of hyperelastic material models. The results were obtained from tensile experiments with parallelly and perpendicularly printed specimens at a loading rate of 10.0 mm/s and used for an initial approximation of the instantaneous material behavior. Data were subsequently modified in order to achieve accurate predictions in FE simulations.

This section describes our calibration of the time-dependent formulation of the hyperelastic Ogden strain energy potential (Eq. 4). For this purpose, optimal values for the hyperelastic parameters *n*, *µ*_*i*_ and *α*_*i*_ and the Prony series parameters *m*, *g*_*k*_ and *τ*_*k*_ had to be determined. In a first approximation, we estimated the instantaneous material behavior based on experiments at a loading rate of 10.0 mm/s, as illustrated in Figure 6. A strain interval from 0 to 1.5 was chosen, and stresses of both print directions were averaged.

To calibrate the hyperelastic material parameters, we applied the Abaqus internal calibration procedure (Dassault Systèmes Simulia Corp, 2014b), which uses a non-linear least-squares method to minimize deviations of the model prediction (Eqs. 5, 6) from experimental stress values (Figure 6). Stretches (also “stretch ratios”) *λ*_*j*_ are related to nominal strains *ε*_*j*_ by *λ*_*j*_ = *ε*_*j*_+1. A three-term (*n* = 3) Ogden model (Eq. 4) showed good accordance with the tensile experiments; the parameter values obtained are given in Table 1. However, as described further below, we found that component simulations based on parameter values *µ*_*i*,10 mm/s_ and α_*i*,10 mm/s_ predicted excessively compliant structural behavior compared to the experimental results. In the absence of biaxial and higher-strain-rate data, we assumed that the actual material behavior was much stiffer than the model predicted. We therefore modified the experimental data by shifting nominal stresses to larger values, as shown in Figure 6. On the basis of an iterative approach of parameter modification and FEA, we propose using the “instantaneous” parameters *µ*_*i,*inst._ and *α*_*i,*inst._ given in Table 1. These were obtained by multiplying experimental nominal stress values by a shifting factor of 7/4 and calibrating the Ogden model to these modified data.

**TABLE 1:** Material parameters for A30 PolyJet elastomer, obtained by fitting a three-term Ogden material model to the original and modified results of uniaxial and planar tensile experiments. The implementation is straightforward in Abaqus/CAE and other nonlinear FE software.

*i*	1	2	3
*µ*_*i,*10_ _mm/s_/MPa	−82.84	36.80	46.85
*α*_*i,*10_ _mm/s_/-	1.26	1.58	0.92
*µ*_*i,*inst._/MPa	−137.40	60.92	77.88
*α*_*i,*inst._/-	1.23	1.55	0.89

**FIGURE 7:**
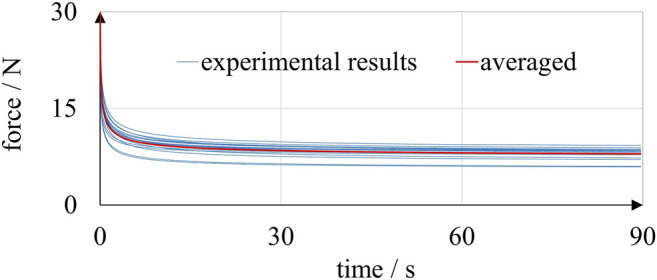
Results of relaxation experiments with linear A30 bellows actuators including data from Dämmer et al. (Dämmer et al., 2019a). Each bellows actuator was loaded twice, and eight different bellows actuators were used in total (blue lines). The averaged results (red line) were used to calibrate a four-term Prony series..

To calibrate the parameters governing the time-dependent decay of stiffness and obtain what we hereafter refer to as our Ogden model, we used the results of relaxation experiments with linear A30 bellows actuators (Dämmer et al., 2019a). The printed bellows actuators were subjected to an enforced displacement of 4.0 mm, which was applied within 0.5 s and held for 89.5 s. The reaction forces were captured as a measure of relaxation; for a detailed description of the experiments, see Dämmer et al. (Dämmer et al., 2019a). To enhance the statistical basis, we repeated the experiments with four additional specimens. In Figure 7, a total of 16 relaxation curves are plotted together with the averaged results (red line).

As shown in Figure 7, the reaction forces dropped from an average of 30.2–7.9 N within the 89.5 s of constant displacement. The marked time dependency of A30 mechanical behavior observed in the experiment supports the findings of previous research (Kundera and Bochnia, 2014; Adamczak and Bochnia, 2016; Reichl and Inman, 2018; Dykstra et al., 2019; Abayazid and Ghajari, 2020). A four-term Prony series was then calibrated by curve-fitting Eq. 7 (*µ*_*i*_ = 1) to the normalized averaged experimental results using a non-linear least-squares method as implemented in Matlab (The MathWorks, Inc.). The Prony parameters *g*_*k*_ and relaxation times *τ*_*k*_ obtained are given in Table 2.

**TABLE 2:** Parameters *g*_*k*_ and relaxation times *τ*_*k*_ for a four-term Prony series to describe the time-dependent decay of stiffness of A30 elastomeric inkjet material. Prony series are supported by nonlinear FE software such as Abaqus/CAE.

*k*	1	2	3	4
*g*_*k*_	0.6293	0.0440	0.0980	0.0403
*τ*_*k*_/s	0.4327	0.4349	3.3484	26.7024

In order to assess the accuracy of our Ogden model, we compared the results of experiments and FE simulations using our model and recently published models. Experimental data was obtained from the bellows relaxation experiment summarized in Figure 7, and corresponding FE simulations were set up.

**FIGURE 8:**
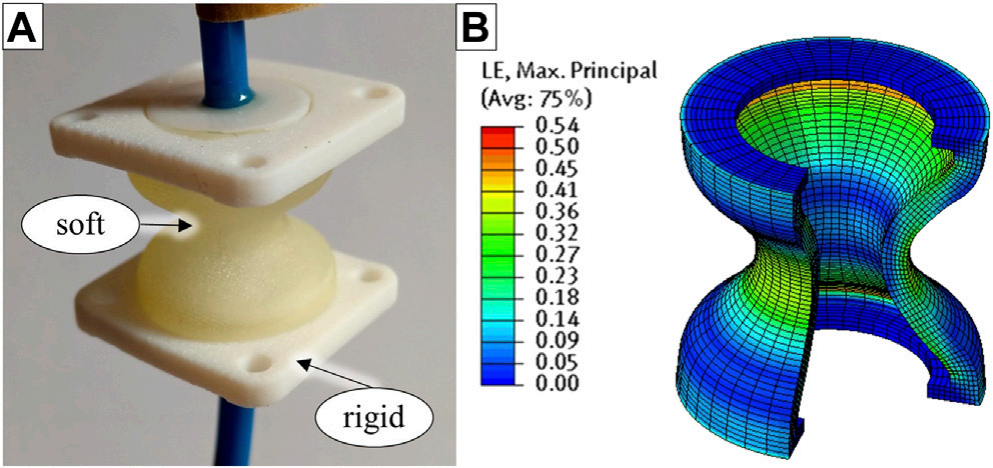
Structural simulation of a bellows relaxation experiment. The inkjet-printed specimens consist of rigid top and bottom flanges and a soft bellows structure **(A)**. In the corresponding FE model, the soft bellows structure is modeled under consideration of its rotational symmetry, and flanges are represented by appropriate boundary conditions **(B)**.

Figure 8A shows one of the inkjet-printed linear bellows actuators, which consists of an elastomeric bellows structure and rigid top and bottom flanges for load application. A cross-sectional sketch of the bellows geometry was imported into the axisymmetric modeling space of the Abaqus/CAE environment, and the bellows geometry was meshed using 396 axisymmetric quadrilateral elements of type CAX8RH (Dassault Systèmes Simulia Corp, 2014a), as illustrated in Figure 8B. Boundary conditions that prevent any relative displacement were applied to the surface nodes in the flange regions.

**FIGURE 9:**
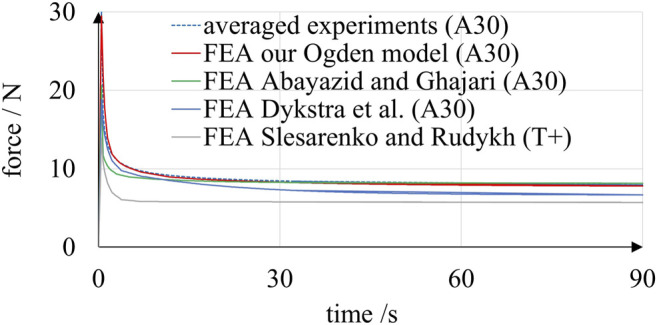
Results of simulated and experimental relaxation of A30 linear bellows actuators. The simulations based on several material models are clearly capable of reproducing the characteristic exponential decay of the experimentally determined reaction force.

A time-dependent displacement of the top flange nodes was applied in accordance with the experimental procedure. Four material models were implemented for comparison: our Ogden model and three recently published models (Slesarenko and Rudykh, 2018; Dykstra et al., 2019; Abayazid and Ghajari, 2020). Abayazid and Ghajari (Abayazid and Ghajari, 2020) provided parameters for a three-term Ogden strain energy potential (Eq. 4, *n* = 3) with five-term Prony series (Eq. 7, *m* = 5). All model parameters for A30 material were provided in (Abayazid and Ghajari, 2020) and can be directly implemented in Abaqus/CAE. Dykstra et al. (Dykstra et al., 2019) published material parameters for a Neo-Hooke strain energy potential with three-term Prony series (Eq. 7, *m* = 3) for A30 material. Assuming fully incompressible behavior (*ν* = 0.5), the instantaneous Young’s modulus *E*_0_ provided can be converted to the parameter *C*_10_ required by Abaqus (Eq. 2) according to *C*_10_ = *E*_0_/6 = 0.542 MPa. Slesarenko and Rudykh (Slesarenko and Rudykh, 2018) published parameters for a two-term Yeoh model to describe the mechanical behavior of T+ material. Comparison of coefficients yields the long-term parameters required as Abaqus input (Eq. 3): *C*_10_ = *µ*/2 = 0.085 MPa and *C*_20_ = *µ*·*α*/4 = 2.55E-3 MPa. Figure 9 plots the simulated reaction forces of the bellows relaxation experiment together with the averaged experimental results. In accordance with the experimental results, the simulated reaction forces show the typical exponential decay regardless of the underlying material model. The results of the simulation are in the expected range, and the strain distribution in the structure is plausible, as illustrated by Figure 8B. As can be seen in Figure 9, FE simulations based on our Ogden model fit the experimental data best, which is plausible, as we tuned our model over several iterations for this purpose. Deviations at 0.5 and 90 s were below 0.7 and 0.1 N, respectively. The experimental results were also matched by simulations based on the material model provided by Abayazid and Ghajari (Abayazid and Ghajari, 2020). At 90 s, a force deviation of only 1.1 N remained, and even closer predictions were observed between 30 and 90 s. However, at 20.6 N the simulated peak force at 0.5 s was relatively low. The reaction forces simulated based on the material model provided by Dykstra et al. (Dykstra et al., 2019) were lower than in the experiments: At 0.5 and 90 s, the simulated reaction forces were 10.1 and 6.7 N, respectively. The FE simulation based on the material model provided by Slesarenko and Rudykh (Slesarenko and Rudykh, 2018) was calibrated to T+ material data. As T+ generally exhibits lower moduli and less pronounced relaxation than A30, which has also been demonstrated by Abayazid and Ghajari (Abayazid and Ghajari, 2020), it is plausible that the corresponding simulations yield lower values than the experiments. Considering known and unknown variations in specimen age, the various origins of the processed data and the particularly complex mechanical behavior of A30, our simulations yielded results with satisfactory accuracy. Our comparison of simulation and experimental results shows that the material parameters provided in this paper and in recent publications (Slesarenko and Rudykh, 2018; Dykstra et al., 2019; Abayazid and Ghajari, 2020) can be valuable in the development of elastomeric PolyJet components. Material model parameters for the hyperelastic Ogden model with Prony series are provided in Tables 1 and 2 and can be directly entered in Abaqus/CAE, which is a typical software for nonlinear FE problems. Other nonlinear FEM software like Ansys (ANSYS, Inc.) and different versions of the Nastran solver also support this type of material model.

## Simulation of Rotary Bellows Deformation Behavior

FE simulations were performed in order to investigate the structural behavior of the rotary bellows actuator presented in *Actuator Design* and to verify the material model derived in *Modeling the Mechanical Behavior of Inkjet-Printed Elastomers* in a real robotic actuation system. This section presents a description of the FE model, simulation results, setup and procedure of the verification experiment and a final comparison.

### Finite Element Model

**FIGURE 10:**
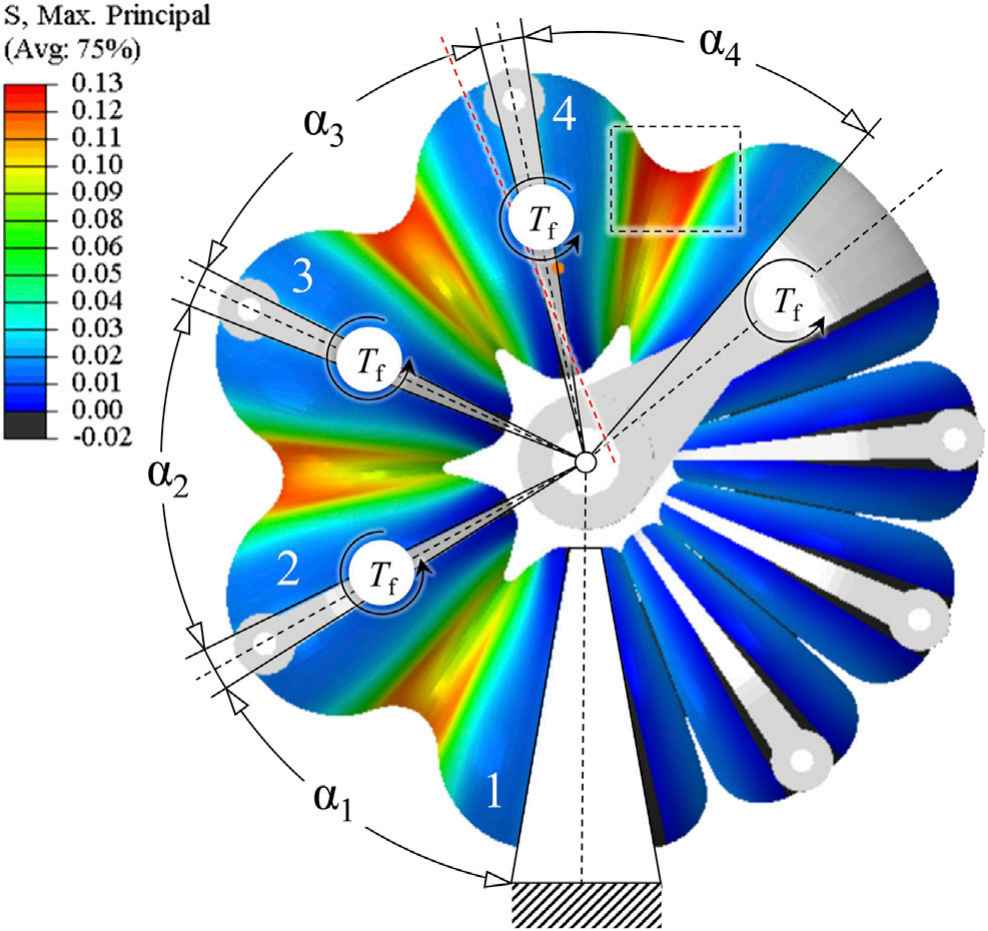
FE model of a rotary bellows actuator. The drive shaft with the movable flange and the frames that connect the bellows segments are idealized to be completely rigid. The bellows segments are modeled as deformable solids with hyperviscoelastic properties. A pressure load was applied to all inner surfaces of the left bellows chamber, and torques T_f_ were applied to flanges and drive shaft to account for frictional effects. Evaluation of the maximum principal stress suggests that structural failure is most likely to occur at the inner diameter of the fourth bellows segment (dashed rectangle).

The FE model used to simulate the deformation behavior of the rotary bellows is explained in Figure 10, which shows the actuator model with example deformation and stress distribution due to pressurization of the left bellows chamber. The model assembly was built from 3D models of the bellows segments, the frames that connect them and the drive shaft with the movable flange that connects the two bellows chambers. The housing of the actuator and the roller guide system were represented by appropriate boundary conditions. Deformable properties were assigned to the bellows segments in accordance with the hyperviscoelastic material model presented above, while the frames and the drive shaft were modeled as being completely rigid. Boundary conditions were applied to the frames and drive shaft to allow only rotation about the center axis. Due to symmetry, only the upper half of these parts was considered; accordingly, all nodes on the virtual slicing plane were restricted in their degrees of freedom.

Pressure loads were applied to the inner surfaces of the left bellows chamber. To account for frictional effects, torque loads T_f_ were applied to each frame of the pressurized chamber and the drive shaft. In Figure 10, a color scale indicates the magnitudes of maximum principal stress. The fourth bellows segment is subjected to the highest loads because frictional torques accumulate toward the movable flange. Hence, structural failure is most likely at the inner ring of the fourth bellows segment (dashed rectangle). Conversely, in a frictionless simulation setup, cyclic symmetry applies for loads and geometry, and stress distribution is equal for each bellows segment.

### Finite Element Simulation

**FIGURE 11:**
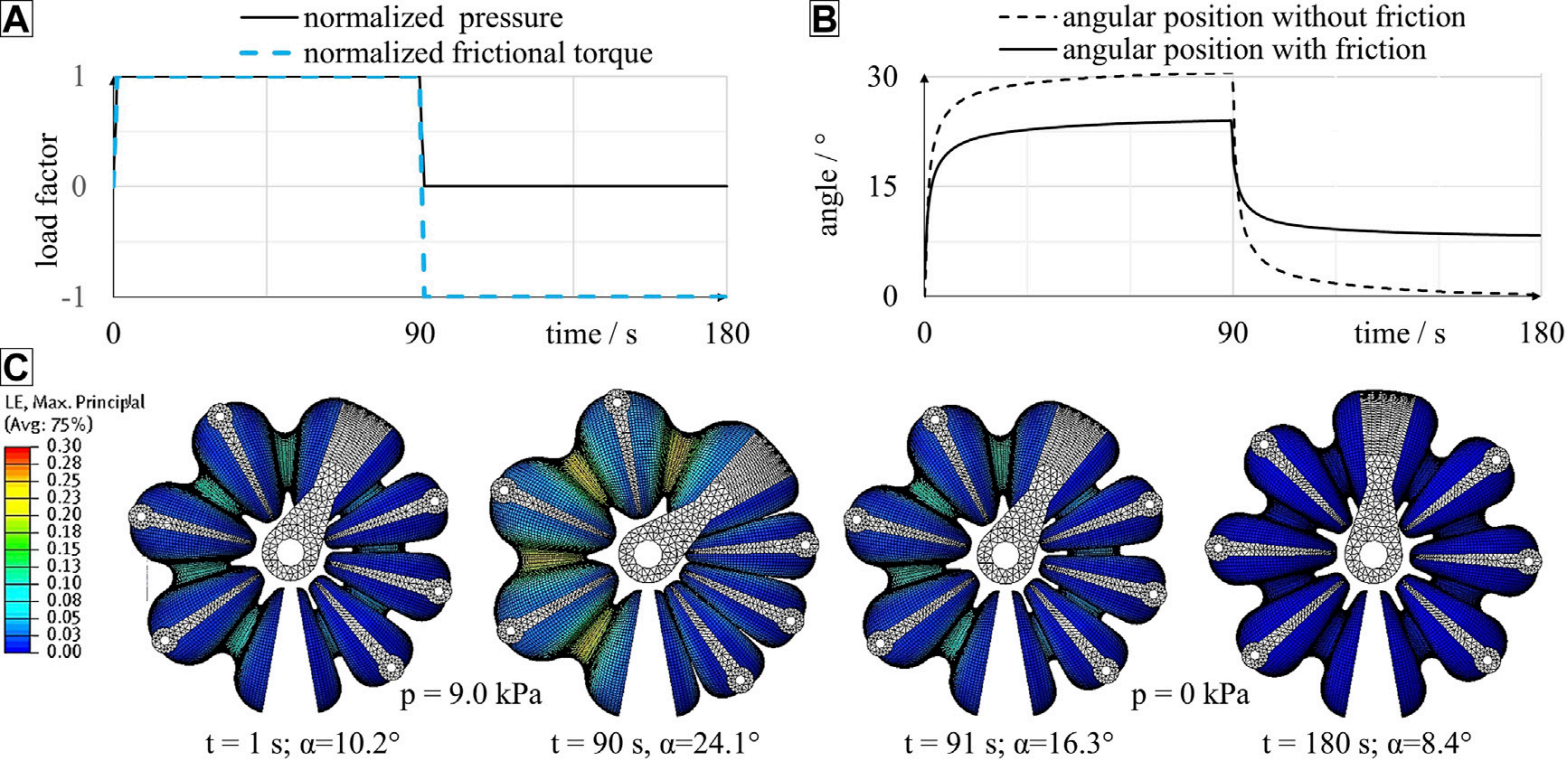
FEA of a novel inkjet-printed rotary bellows actuator including the definition of loads **(A)**, evaluation of time-dependent angular deflection **(B)** and structural deformation **(C)**. As shown in **(A)**, a pressure load and (optional) torque loads were defined to simulate a 90 s-interval of pressurization followed by 90 s of zero pressure. As shown in **(B)**, angular deflection is considerably time-dependent during phases of constant pressure. The consideration of torque loads causes a decrease in maximum angle at 90 s and an increase in residual angle at 180 s. As shown in **(C)**, significant time-dependency can also be observed by evaluation of strain distribution and structural deformation. In this simulation run pressure loads of 9.0 kPa and torque loads of 1.8 Nmm were considered.

Using the FE model described above in combination with the hyperviscoelastic material model, described as our Ogden model in *Modeling the Mechanical Behavior of Inkjet-Printed Elastomers*, we simulated the rotary bellows actuator’s time-dependent deformation behavior if subjected to successive application and release of pressure. In Figure 11A, the normalized load magnitudes are plotted over the simulated time period of 180 s. Pressure loads were ramped up linearly and held constant until 90 s of simulation time. Pressure was then linearly reduced to ambient level and held constant until the end of simulation. In the actuator, frictional torques generally act against the direction of rotation. Torque loads in the simulation were therefore defined as positive and negative during phases of pressurization and zero pressure, respectively. Changes in torque loads were interpolated linearly and applied simultaneously to changes of pressure loads. In Figure 11B, the simulated time-dependent angular deflection during a 9.0 kPa-pressure cycle is plotted. Changes in pressure were applied at a rate of 9.0 kPa/s, and the torque loads applied were 1.8 Nmm each. For the period of positive pressure, the actuator rotated clockwise in the simulation. As soon as pressure was released at 90 s, the direction of rotation was reversed. The angular deflection during periods of constant pressure was clearly time-dependent and the characteristic shape of exponential decay, as previously found in the relaxation experiments (Figure 7), was also observed in this load-controlled simulation. In order to investigate frictional effects, the underlying simulation was repeated without frictional torques and the results plotted for comparison. As shown in Figure 11B, the omission of torque loads led to an increase in maximum angle at 90 s and a decrease in the residual angle at 180 s. While in this example the residual angle was close to zero (0.3°) for the frictionless setup, it amounted to 8.4° for the setup considering frictional torque loads. In Figure 11C, simulation results of the bellows actuators deformation and strain distribution are plotted at various time points of the simulation. Maximum logarithmic principal strains are indicated using the same color scale in all graphs. At 1 s, the target pressure of 9.0 kPa is reached in the left chamber; color gradients indicate the presence of strains, especially at the inner regions of the bellows segments. As a result of internal pressure, the structure deforms, and an angular clockwise deflection of 10.2° is visible. At 90 s, the bellows structure has deformed significantly. While the left pressurized chamber has expanded and caused clockwise rotation of the actuator by 24.1°, the right, pressure-free chamber is compressed. At 91 s, pressure has returned to zero, and the angular position has decreased rapidly. In this example, however, the actuator remains deflected by 16.3° from its initial position. At 180 s, the actuator has returned close to its initial state. A residual angular deflection of 8.4° remains although no pressure has been applied in the last 89 s. Our simulation results presented in Figure 11 predict a pronounced time-dependent behavior of the elastomeric structure of the rotary bellows actuator introduced in *Actuator Design* and moreover reveal the importance of considering friction.

### Verification Experiment

**FIGURE 12:**
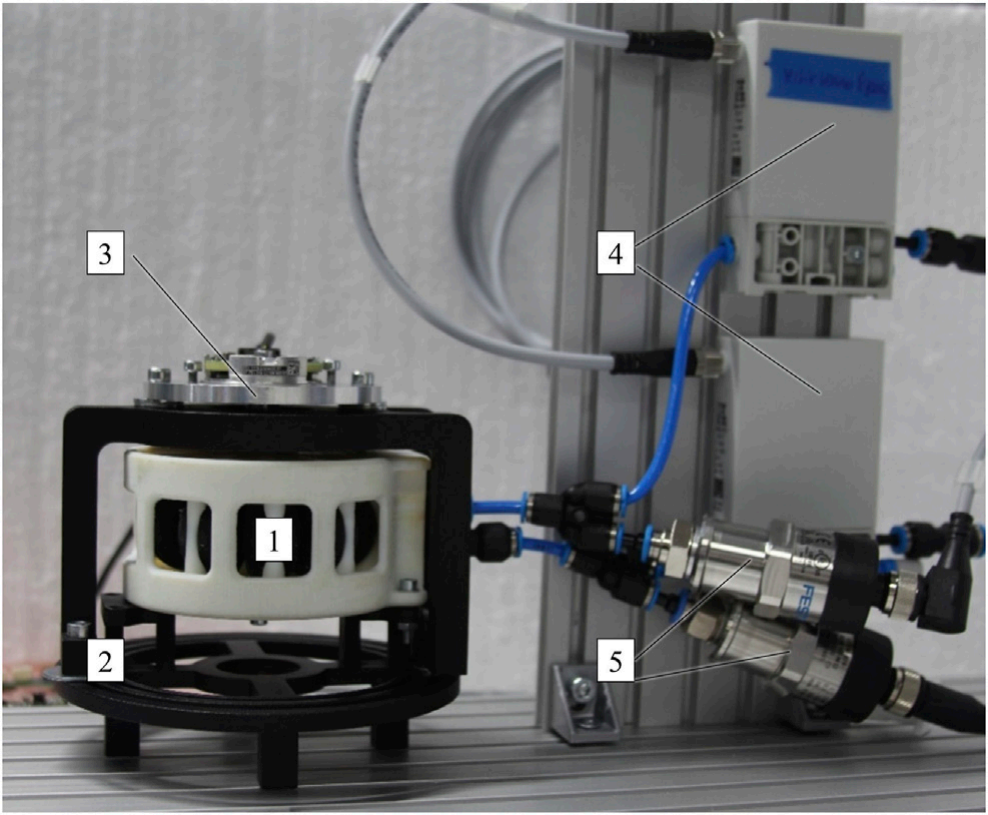
Experimental setup for investigating the time-dependent structural behavior of pneumatic rotary bellows actuators.

As shown in Figure 12, a rotary bellows actuator 1) was mounted on a carrier frame 2) together with a Haidenhain ECI 11118 EnDat 22 absolute encoder 3). Two Festo VEAB proportional valves 4) with a pressure range of 0.5–100.0 kPa were connected to the bellows chambers. The VEAB valves provided an integrated controller which, in combination with piezo technology, allowed fast and precise setting of the chamber pressures. Two Festo SPTW pressure sensors 5) with a pressure range of −100.0 to 100.0 kPa were used to measure chamber pressures. A dSPACE (dSPACE GmbH) real-time platform with a DS1005 processor card was employed to control the valves and capture the pressure sensor data. The processor card was programmed *via* Matlab/Simulink (The MathWorks, Inc.). In accordance with Figure 11A, linear reference trajectories of the target pressure were implemented in Simulink and sent *via* the real-time interface.

One of the actuator’s chambers was pressure-controlled while the counteracting chamber was vented. A relative chamber pressure of 1.5 kPa was applied and held constant. After 90 s the chamber was vented completely for 90 s to allow the actuator to return to its initial position. In total, six pressure levels ranging from 1.5 to 9.0 kPa were applied in 1.5 kPa steps and the procedure was repeated five times at each pressure level. All changes in pressure were applied at a rate of 9.0 kPa/s. Two pristine actuators (i.e., four actuator chambers) were subjected to the described loadings. The actuators used had been assembled carefully and not subjected to any significant prestress.

### Experimental Results

**FIGURE 13:**
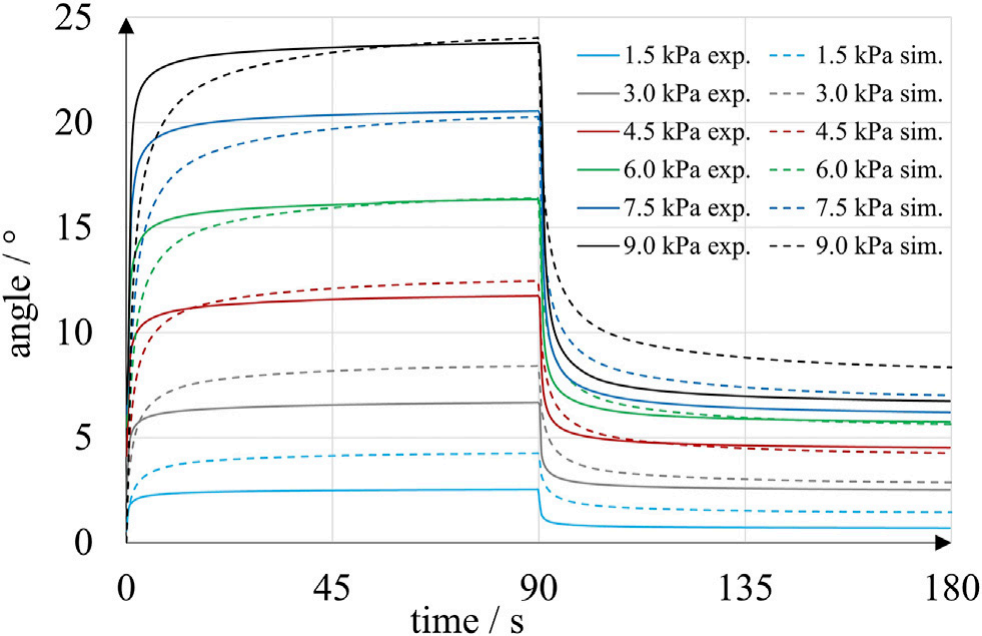
Time-dependent angular position of inkjet-printed rotary bellows actuators in experiments (solid lines) and simulation (dashed lines). Each solid curve is an average of five experiments with four bellows chambers at a particular pressure level.

Figure 13 compares the results of experiments and simulations: Each solid curve is an average of 20 load-unload cycles. In the experiment, the actuators rotated clockwise for the first 90 s at decreasing speed. As soon as pressure was released at 90 s, the direction of rotation reversed, and the measured angular position approached an almost flat plateau. An increase in pressure level generally corresponded to an increase in both maximum angular deflection at 90 s and final angle at 180 s. Even the smallest pressure of 1.5 kPa caused rotation of the bellows actuators. The averaged maximum angles reached within the experiment ranged from 2.5 at 1.5 kPa to 23.8° at 9.0 kPa. A rapid increase in pressure led to immediate rotation of the bellows actuator. Between 0 s and 90 s, all curves follow a degressive trend, which indicates pronounced time-dependent behavior of the bellows structure. Typically, almost 90% of the final deflection was reached after 3 s. At 90 s, the slopes of the curves have decreased significantly but remain positive, which suggests that extension of the load phase would result in an additional increase in angular position. Venting the chambers at 90 s resulted in an instantaneous decrease in angular deflection. During the venting phase the curves follow a regressive trend. Though the chamber pressures quickly returned to ambient level within less than 1 s, the angular positions measured decreased slowly and returned almost to 0° within the experimental time.

Remarkably, curves that were averaged at higher pressures, settled at larger final angles. For example, experiments at 9.0 kPa and at 1.5 kPa resulted in averaged final angular deflections of 6.7° and 0.7°. However, the final angles also increased in successive loadings of the same magnitude as a result of the specific sequence of load-unload cycles. According to simulations of the blocked actuator, torques of up to 20 Nmm were created at 0° angular deflection and 9.0 kPa pressure.

### Comparison of Experiment and Simulation

The experiments described above were simulated as described in *Finite Element Simulation*. Pressure magnitudes between 1.5 and 9.0 kPa were applied in accordance with the experimental procedure, and frictional torques were applied as illustrated in Figure 10. Magnitudes of frictional torques were assumed proportional to the applied pressures, with a proportionality constant of 20 Nmm/bar. For example, in simulations corresponding to experiments with 9.0 kPa, torques of 1.8 Nmm were applied to each of the flanges and the drive shaft of the actuator. The simulation results for all pressures and torque levels are plotted in Figure 13 and indicate a strong time dependency of the angular position, very similar to the behavior observed in the corresponding experiments. For pressures above 3.0 kPa, the simulated angles lagged behind the experimental values during the first seconds of pressurization. At 90 s, good experimental-simulative correlation with small deviations of 0.2°, 0.3° and 0.1° was observed for the three higher pressure levels of 9.0, 7.5 and 6.0 kPa, respectively. For the lower pressure levels of 4.5, 3.0 and 1.5 kPa, larger deviations of 0.7°, 1.7° and 1.7° occurred, respectively. Venting the pressurized chamber at 90 s initiated an exponential decay of angular deflection that was more rapid in the experiments than in the corresponding simulations. The largest deviation in final angle at 180 s was observed at 9.0 kPa, where the simulation predicted an angle of 8.4°, which is 1.6° greater than the experimentally determined value. Experiment and simulation are in closer agreement at intermediate pressures of 3.0, 4.5 and 6.0 kPa, where the deviations amount to 0.7°, 0.3° and 0.3°, respectively. We found that, by shifting the applied levels of torque in the pressurization and venting phase, the simulated angles were also shifted and good correlations of maximum and final angles at 90 and 180 s were achieved. Due to the absence of external load torques in our experiments, the unknown frictional torques have a relatively large effect on the behavior of the actuator. Future experiments should consider defined external load torques and modeling of friction should be improved.

## Conclusion

We designed highly integrated rotary bellows actuators for three-dimensional (3D) inkjet printing and demonstrated that the time-dependent behavior of their elastomeric bellows structure can be estimated by Finite Element (FE) simulations. The actuators presented here are a first foray into the unexplored field of multi-material rotary bellows actuators. Various photocurable thermoset inks that exhibit rigid or elastomer-like properties after curing were used. The cylindrical actuator housing measures 80 mm in diameter and 44 mm in height. Functionality and durability of the design presented were proven by extensive pressure loadings at various pressure magnitudes. At a pressure of 9.0 kPa, the actuators produce torques of up to 20 Nmm and can reach angles close to 24°. Larger pressures can be applied to increase torque and angular displacement but reduce the lifespan of the bellows structure. Our results build on previous research in the field of printed bellows actuators (Dämmer et al., 2019a; Dämmer et al., 2019b) and rotary bellows devices (e.g., Bousso, 1970; Stoll and Schlötzer, 2008; Kaminski and Knubben, 2012). Transferring the underlying functional principle to state-of-the-art 3D inkjet printing allowed us to integrate the three main actuator components—the housings, two deformable bellows chambers and the drive shaft—into a single multi-material part. Despite using a sophisticated simulation-based bellows design, the maximum torque and actuation range of the actuators are still significantly too low for use in commercial robots which is due to the limited mechanical properties of inkjet-printed elastomers. Nevertheless, 3D inkjet printing is considered an important bridging technology and has taken on a significant role in soft robotics and multi-material prototyping as it allows researchers for the manufacturing of conceptual multi-material components with unmatched complexity. Our results confirm the findings of previous reports of the time-dependent mechanical behavior of inkjet-printed elastomers (Kundera and Bochnia, 2014; Adamczak and Bochnia, 2016; Reichl and Inman, 2018) and show similar behavior in complex bellows structures printed from a photocurable elastomer with a Shore A hardness of 30. We analyzed previously published relaxation experiments with linear bellows actuators (Dämmer et al., 2019a), strengthened the experimental basis and found that, on average, reaction forces decreased by 74% within a 89.5 s duration of constant elongation. Further, we measured the angular deflection of our novel rotary bellows actuators during constant pressure intervals of 90 s. We found that deflection was significantly time-dependent and 90% of the final deflection was typically reached within the first 3 s after a pressure step. To describe the instantaneous material behavior of the inkjet-printed elastomer, we calibrated a three-term Ogden strain energy potential using uniaxial and planar tensile experiment data at three different loading rates. To account for time dependency, the moduli of the Ogden model were expressed by means of a (time-dependent) Prony series that was calibrated to a bellows relaxation experiment as shown by Dämmer et al. (Dämmer et al., 2019a). We have demonstrated that the time-dependent structural behavior of inkjet-printed elastomers can be estimated by means of non-linear FE simulations using hyperviscoelastic material models. To evaluate the accuracy of the calibrated material model, we performed FE simulations of the bellows relaxation experiment and compared them to similar simulations based on models from recent publications (Slesarenko and Rudykh, 2018; Dykstra et al., 2019; Abayazid and Ghajari, 2020). All models predicted a characteristic time-dependent decay of reaction force in accordance with the experimental data. Modification of the model we calibrated yielded good accordance between experiment and simulation, with remaining deviations amounting to less than 2%. We then used this modified material model for structural simulation of our novel rotary actuator. Two subsequent load steps were defined to simulate the time-dependent angular deflection of the rotary actuator due to a 90 s pressurization and a 90 s venting phase of one bellows chamber. We found that experiment and simulation were in close agreement when frictional effects were considered. Based on our results from experiments and simulations, we conclude that ignoring time dependency in the material model will lead to poor approximation of the structural behavior, especially for relatively dynamic applications involving time spans under 30 s. The methodology presented is valid for all kinds of elastomers and is not limited to 3D inkjet-printing technology or robotics. The material model parameters we provided can be used for direct implementation in Abaqus/CAE and other FE software to enable the simulation-based development of elastomeric inkjet-printed parts for mechanically stressed prototypes. Future work will address the fatigue behavior of the actuators and demonstrate their application in an additively manufactured robotic system. In the longer term, customizability of the presented design should be demonstrated and automated by means of simulation-driven design processes. As soon as more resilient materials are developed, the actuators shown here should be manufactured using these materials.

## Data Availability

The original contributions presented in the study are included in the article/Supplementary Material, further inquiries can be directed to the corresponding author.
