# Corrigendum: Design of an Inkjet-Printed Rotary Bellows Actuator and Simulation of its Time-Dependent Deformation Behavior

**DOI:** 10.3389/frobt.2021.729549

**Published:** 2021-07-09

**Authors:** Gabriel Dämmer, Michael Lackner, Sonja Laicher, Rüdiger Neumann, Zoltán Major

**Affiliations:** ^1^ Institute of Polymer Product Engineering, Johannes Kepler University Linz, Linz, Austria; ^2^ Advanced Development Control and Robotics, Festo SE and Co. KG, Esslingen, Germany

**Keywords:** bellow actuator, printed robotics, design for additive manufacture, multi-material 3D printing, soft pneumatic actuator, time-dependent materials, printed elastomer, PolyJet elastomers

In the original article, there was a mistake in Figure 13 as published. The angles were too small by a factor of 10 in Figure 13. However, the angular values in the text and all other figures are correct. The corrected Figure 13 appears below.

**FIGURE 13:**
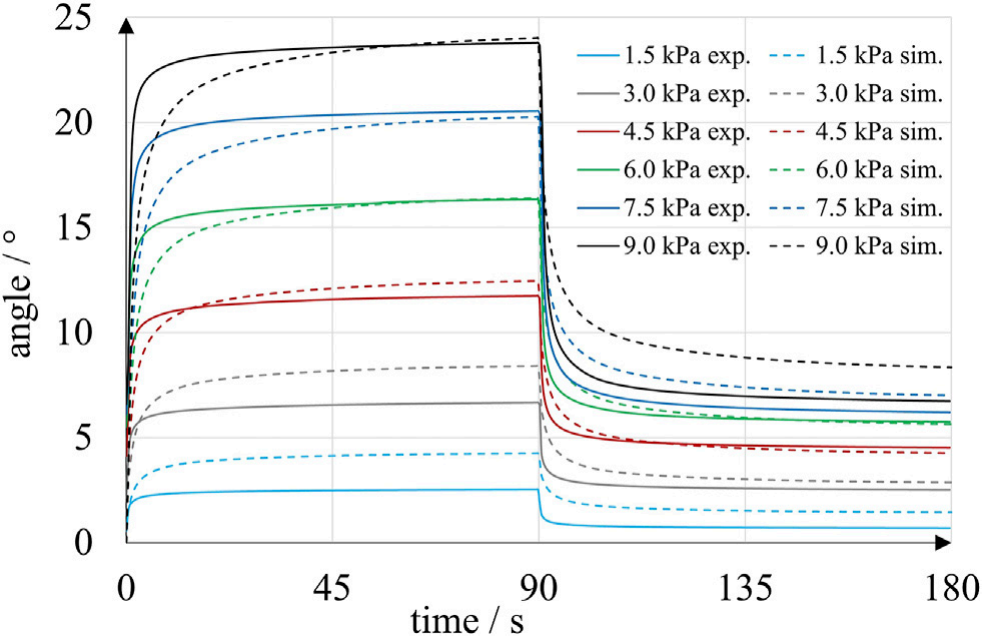
Time-dependent angular position of inkjet-printed rotary bellows actuators in experiments (solid lines) and simulation (dashed lines). Each solid curve is an average of five experiments with four bellows chambers at a particular pressure level.

The authors apologize for this error and state that this does not change the scientific conclusions of the article in any way. The original article has been updated.

